# CompRet: a comprehensive recommendation framework for chemical synthesis planning with algorithmic enumeration

**DOI:** 10.1186/s13321-020-00452-5

**Published:** 2020-09-01

**Authors:** Ryosuke Shibukawa, Shoichi Ishida, Kazuki Yoshizoe, Kunihiro Wasa, Kiyosei Takasu, Yasushi Okuno, Kei Terayama, Koji Tsuda

**Affiliations:** 1 Graduate School of Frontier Sciences, The University of Tokyo, Kashiwa, Chiba, Japan; 2 Graduate School of Pharmaceutical Sciences, Kyoto University, Sakyo-ku, 606-8501 Kyoto, Japan; 3 RIKEN Center for Advanced Intelligence Project, Tokyo, Japan; 4 Toyohashi University of Technology, Aichi, Japan; 5 Graduate School of Medicine, Kyoto University, Kyoto, Japan; 6 Medical Sciences Innovation Hub Program, RIKEN, Kanagawa, Japan; 7 Graduate School of Medical Life Science, Yokohama City University, Kanagawa, Japan; 8 Research and Services Division of Materials Data and Integrated System, National Institute for Materials Science, Kyoto, Japan

**Keywords:** Retosynthesis, Enumeration, Computer-assisted synthesis planning

## Abstract

In computer-assisted synthesis planning (CASP) programs, providing as many chemical synthetic routes as possible is essential for considering optimal and alternative routes in a chemical reaction network. As the majority of CASP programs have been designed to provide one or a few optimal routes, it is likely that the desired one will not be included. To avoid this, an exact algorithm that lists possible synthetic routes within the chemical reaction network is required, alongside a recommendation of synthetic routes that meet specified criteria based on the chemist’s objectives. Herein, we propose a chemical-reaction-network-based synthetic route recommendation framework called “CompRet” with a mathematically guaranteed enumeration algorithm. In a preliminary experiment, CompRet was shown to successfully provide alternative routes for a known antihistaminic drug, cetirizine. CompRet is expected to promote desirable enumeration-based chemical synthesis searches and aid the development of an interactive CASP framework for chemists.

## Introduction

Since the 1960s, several researchers have proposed computer-assisted chemical synthetic route designs. Various computer-assisted synthesis planning (CASP) programs have been developed to assist synthetic organic chemists in their work [1–3]. While expert systems and knowledge-based programs were the primary focus of CASP during the early stages [4–8], recent breakthroughs in the field of deep learning and widespread availability of reaction datasets have accelerated its development [9–17]. In particular, data-driven approaches have received attention across research fields [18–21]. These approaches for multi-step synthesis planning have shown outstanding performance at every stage, and more recently, they have provided realistic and preferable synthetic routes.

The pioneers of CASP, Corey and Wipke, stated the following requirements related to the above strategy in their paper [2]: the program needs to provide as many useful routes as possible, chemists can decide the depth of search or analysis of the synthetic route, and the given routes are evaluated by the chemists. As discussed above, several CASP approaches have been developed; however, the majority of them have aimed to directly obtain the optimal chemical synthetic route rather than attempting to provide multiple route candidates. According to Corey [2], examining as many useful chemical synthetic routes as possible is an essential part of retrosynthetic analysis. It is well known that evaluation criteria used for the presented synthetic routes depend on the chemist’s situation, objectives, and/or needs [22], such as the early-stage derivatization of hits, optimization of lead compounds, or large-scale synthesis of drug candidates. Thus, a desirable framework should provide as many useful routes as possible under specific conditions (e.g., room and high temperatures) and choose multiple reliable routes based on given situations (e.g., drug discovery or drug development stages).

As a framework for providing multiple reliable routes, Kowalik et al. have developed a promising approach using the Network of Organic Chemistry (NOC) [23–27] and an enumeration algorithm of possible synthetic routes [28]. The NOC consists of all possible molecules and reactions that represent links from reactants to products. [27] The reactions are practically represented as templates that include the conditional/contextual rules of chemistry. Additionally, they implemented a recommendation system of multiple synthetic routes for a target molecule as follows [28]. Firstly, they extracted the network of molecules and reactions (chemical reaction network) related to the target from the NOC. Secondly, they enumerated all possible synthetic routes from the chemical reaction network, and then, selected promising candidate routes. Although they showed a vast number of synthetic routes for some molecules and presented realistic solutions, their approach has two potential issues: the NOC is very large, and thus, uneconomical for obtaining optimal routes for a specific target molecule, and the enumeration algorithm does not always provide all possible routes. Hence, an efficient algorithm for constructing a chemical reaction network is required for practical application. Further, an exact enumeration algorithm without loss or duplication is needed for practical usage and finding reliable alternative routes.

In this study, we propose a CASP framework called “CompRet,” which enumerates possible synthetic routes using a novel enumeration algorithm with a theoretical guarantee, and then selects useful routes based on several score functions. CompRet implements the following three steps to recommend synthetic routes: (1) constructing a chemical reaction network based on the depth-first proof number search (DFPN) and template-based retrosynthesis [29, 30], without a large chemical reaction network such as the NOC, (2) enumerating all synthetic routes from the network using a novel algorithm, and (3) recommending multiple synthetic routes by developing a naive visualization method and simple score functions. DFPN was initially developed by Nagai et al. in the context of artificial intelligence for games such as Shogi and Go [31–33]. In application to CASP, it has shown superior or comparable performance to that of depth-first search or Monte Carlo tree search [34, 35]. Therefore, DFPN was adopted to construct the chemical reaction network proposed herein. The number of possible synthetic routes provided by the enumeration may reach or exceed several millions. As it would be impossible for chemists to manually examine all of them, several score functions and a visualization method have been introduced into the framework to simplify the process.

Here, we report the development of CompRet and mathematically prove the completeness and soundness of the proposed enumeration algorithm, which can precisely enumerate all synthetic routes from a given constructed chemical reaction network. To demonstrate the approach, possible synthetic routes were found for cetirizine, an antihistaminic drug. In addition to sorting routes by scores, an embedding method to obtain an overview of millions of synthetic routes by defining a route fingerprint was attempted.

## Method

**Fig. 1:**
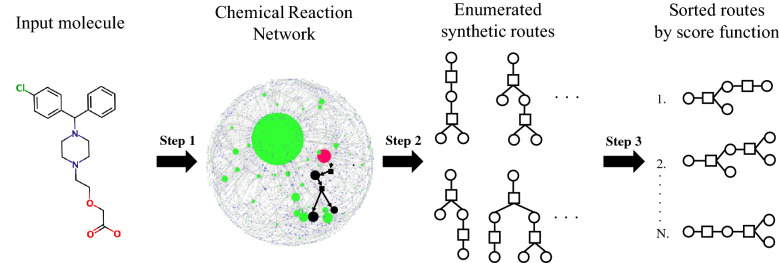
Illustration of CompRet’s overall processes. Step 1: Construction of a chemical reaction network. Step 2: Enumeration of synthetic routes. Step 3: Ranking synthetic routes based on the scoring function

Compret consists of three steps (Fig. 1). Each step is described as follows.

### Construction of chemical reaction network

**Fig. 2:**
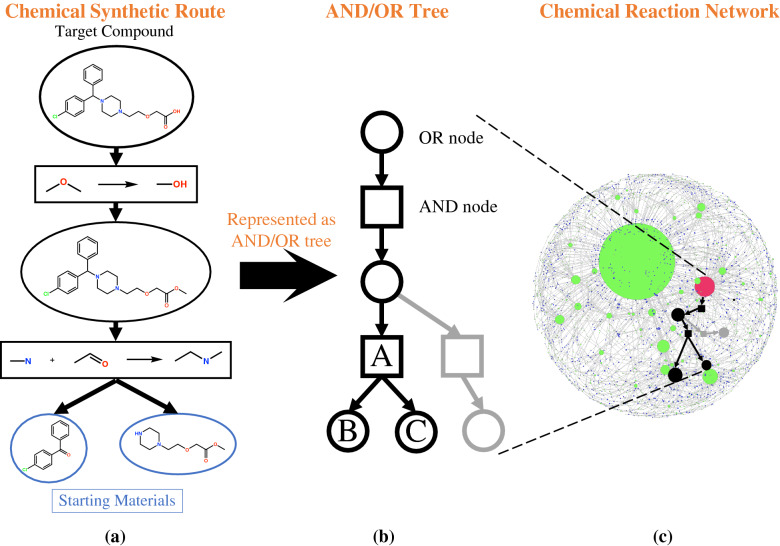
AND/OR representation of synthetic routes.** a **Example of a single synthetic route for cetirizine. Retrosynthetic computation is performed recursively from the topmost node (target) until it reaches to the starting materials at the bottom.** b** The synthetic route can be represented as an AND/OR tree in which the OR and AND nodes denote molecules and reactions, respectively.** c** A chemical reaction network ideally consists of all possible synthetic routes to a target (red sphere) molecule

A synthetic route for a target molecule can be represented as a tree-like structure in which molecule nodes (circles) and chemical reactions (rectangles) appear alternately, as shown in Fig. 2(a). In order to make a route feasible, the end molecule nodes (molecules in the blue circles in Fig. 2(a)) must consist of starting materials (e.g., commercially available molecules), and each synthesis step should be reasonable [29, 30].

The chemical reaction network of a target molecule is typically large and can efficiently express (ideally all) possible synthetic routes to the target represented by the molecule and reaction nodes [27, 36]. In this study, we represent synthetic routes and chemical reaction networks as AND/OR trees, as shown in Fig. 2(b), to efficiently construct chemical reaction networks and precisely perform enumeration (see Additonal file 1: for details on the AND/OR tree and chemical reaction network). The synthetic route in Fig. 2(a) is represented as the black route in Fig. 2(b) by expressing the molecule and reaction nodes as OR and AND nodes, respectively. The gray route in Fig. 2(b) shows another route to synthesize the same reactant. In an AND/OR tree, a molecule is represented as an OR node because either black “OR” gray routes are available to synthesize the same reactant, as shown in Fig. 2(b). On the other hand, a reaction is represented as an AND node because all the reactants (e.g., B “AND” C OR nodes in Fig. 2(b)) of the reaction (AND node A in Fig. 2(b)) are required to synthesize the product. Merging the molecule and reaction nodes that appear in different synthetic routes in this manner (Fig. 2(b)) enables an efficient representation of a large number of routes as a chemical reaction network (Fig. 2(c)).

CompRet efficiently constructs the chemical reaction network for a given target molecule based on DFPN, a search method based on the AND/OR tree using proof and disproof numbers for each node (see Additonal file 1: for details on the DFPN algorithm). To design a synthetic route, reaction templates are applied to a target to transform it into reactants. For the retrosynthetic computation, Reactor version 20.11.0 (ChemAxon [37]), which can consider stereochemistry in reaction templates, was used. The relevance of the transformed reactants was checked by computing the product of the template and the reactants. By recursively performing this transformation according to the DFPN algorithm, all possible synthetic routes for a target molecule can be obtained upon reaching the preset maximum depth *md*. The algorithm can design longer synthetic routes with a larger *md* value. Furthermore, CompRet repeatedly searches for a new route and merges it into a chemical reaction network (see the section 1 and 2, and Fig. S1 in Additional file 1: for details on the construction algorithm).

### Enumeration algorithm

**Fig. 3:**
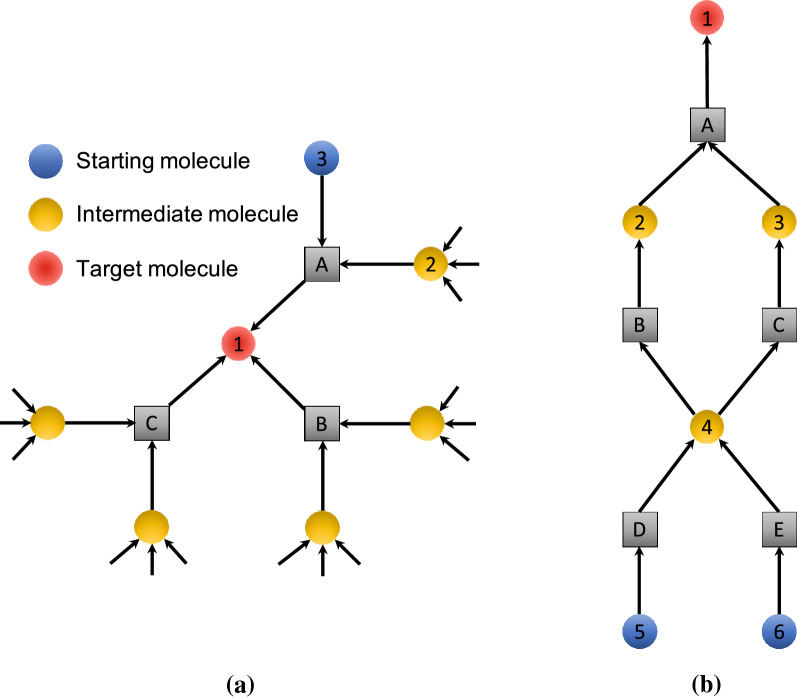
Example of the local structure of a chemical reaction network to illustrate the method used to calculate the number of synthetic routes. **a** Ideal structure of a network for which the naive method can count the exact number of synthetic routes for a target molecule. **b** The naive method cannot count synthetic routes precisely in this case. The number of synthetic routes for the target molecule, *mol*(1).*count*, is calculated as $mol(5).count=1, mol(6).count=1, rxn(D).count=mol(5).count=1, rxn(E).count=mol(6).count=1, mol(4).count=rxn(D).count+rxn(E).count=2$, and finally $mol(a).count=4$, while the true number of synthetic routes is 2

Enumerating all synthetic routes in the chemical reaction network of a given target may appear to be a simple problem, as described in the literature reported by Kowalik et al [28]. For example, in Fig. 3(a), the target (molecule 1) can be synthesized via any one of the reactions A, B, or C. Here, we consider how to count all possible synthetic routes. Synthetic routes for the target can be counted using the following equation:

1 $$\begin{aligned} mol(1).count = rxn(A).count+rxn(B).count+rxn(C).count, \end{aligned}$$

where *mol*(1) and *rxn*(*X*).*count* denote the number of routes to synthesize the molecule 1 and the number of routes that use the reaction *X*, respectively. On the other hand, the number of ways to prepare reactants for a reaction is calculated as follows. In the case of *A*,

2 $$\begin{aligned} rxn(A).count = mol(2).count \times mol(3).count. \end{aligned}$$

These calculations are performed recursively until starting materials for which *mol*.*count* is assigned one (e.g., $mol(3).count=1$).

**Fig. 4:**
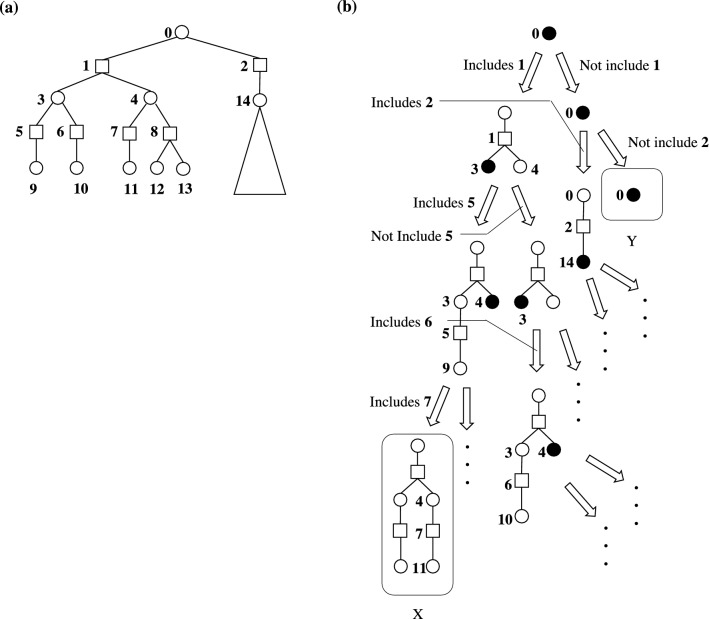
Illustration of the enumeration algorithm. **a** An example of an input chemical reaction network. **b** Visualized procedures of the enumeration algorithm. The black filled circle is a node to focus on. X is a terminal state where a synthetic route is constructed. Y is also a terminal state where there is no node to focus on because all child nodes (1, 2) are already checked

However, as mentioned in the literature [28], this procedure does not count the exact number of synthetic routes because it assumes that each reactant is synthesized independently. In the actual network, a single molecule can act as a reactant for several reactions; for example, the molecule 4 in Fig. 3(b). Note that both molecules 2 and 3 are required for the reaction *A*. The network depicted in Fig. 3(b) includes only two synthetic routes for the molecule 1, i.e., a choice of the reactions *D* or *E* for synthesizing the molecule 4, while the number of synthetic routes is calculated to be 4 according to the above equations. Therefore, we propose an enumeration algorithm that extracts all possible routes in a network by considering “joined nodes”, as shown in Fig. 3(b). A brief description of the enumeration algorithm is given in Fig. 4. The details of the enumeration algorithm are described in the Additonal file 1.

### Route ranking for recommendation

Three scoring functions were utilized for recommendation from the enumerated synthetic routes: the step-based method (STEP), mean synthetic complexity score (MSCS), and reference route-based method (REF). STEP is a simple method that outputs the longest number of synthesis steps for a given synthetic route. Synthetic complexity score (SCScore) was developed by Coley et al. [12] to evaluate the complexity of the molecule. Like the SCScore, the MSCS for a synthetic route ranges from 0 to 5. As the SCScore of a molecule is directly proportional to the complexity of its synthesis, a molecule with a lower SCScore is preferred in synthesis planning. Here, MSCS is defined as the average of the SCScores of all molecules in a synthetic route. MSCS takes into account the complexity of intermediate molecules. REF is calculated only if a reference synthetic route is given. First, all the molecules that appear in the reference route are extracted and sorted by molecular weight. A list of sorted molecules is similarly prepared for a designed synthetic route. Then, the sum of fingerprint-based similarities between the sorted molecules of the reference and given routes is calculated. The RDKit fingerprint [38, 39] and Tanimoto metrics [40] were employed for this similarity evaluation. If the lengths of the sorted molecule lists differ, REF is set to 0. This score function is designed to find synthetic routes that are slightly different from the reference route, using the same number of intermediate molecules. Smaller STEP and MSCS values indicate a superior route, whereas REF is designed such that a larger value indicates a more desired route.

### Visualization for confirming route distribution

To confirm that the CompRet framework is capable of designing a wide variety of synthetic routes, we developed a simple method to plot the routes in a 2D space by converting a synthetic route into a vector. For the conversion, route fingerprint $\varvec{f}_r$ for a route *r* is defined as

3 $$\begin{aligned} \varvec{f}_r&= \sum _{t \in r} \varvec{f}_p(t), \end{aligned}$$

where *t* is a reaction template and $\varvec{f}_p(t)$ is the structural reaction fingerprint [41] of *t* computed by RDKit [39]. Following the computation of route fingerprints for 3,000 sampled routes, t-SNE embedding [42] was computed using scikit-learn [43].

### Reaction template and building block

Template-based approaches generally require both reaction templates and starting materials. A reaction template is represented as a generalized chemical reaction, and technically represented as a reactive center and the first neighboring atoms and bonds in a reaction. The reaction templates were extracted from 27 million single-step reactions obtained from Reaxys (from 1795 to 2019) [44], following the method used in a previous study. [17] Here, the single-step reactions obtained from Reaxys were filtered on the condition that a reaction has a product and up to three reactants. Five hundred reaction templates were used in the order of occurrence frequency. In total, about 13 million reaction templates were extracted; in the top 500 templates, frequency counts are range from 1,906 to 188,460. Starting materials were defined as commercially available chemical compounds and used as stopping criteria for DFPN. For these, 157,544 molecules from Enamine building blocks [45] were used.

## Results and discussion

### Proof of enumeration algorithm

The proposed algorithm can enumerate all possible routes without loss or duplication from a given chemical reaction network of a target molecule. To prove this, it is necessary to show that (1) the algorithm outputs only synthetic routes, (2) there are no duplicate outputs, and (3) the algorithm outputs all synthetic routes in a given chemical reaction network. Here, these properties have been proven using the partition method [46, 47], which is widely used for enumeration algorithms, and mathematical induction. Details of these proofs are given in the Additonal file 1. The properties (1), (2), and (3) are shown in Theorem 4.1, Lemma 4.1, and Theorem 4.2, respectively. The algorithm described in the literature reported by Kowalik et al. [28] cannot count the number of synthetic routes accurately, as discussed in the Methods section. On the other hand, the proposed enumeration algorithm outputs all synthetic routes without loss or duplication, based on the idea of the “prohibited list” (variable *P*) and related procedures in Algorithm S3.

### Route enumeration for Heifets’ benchmark

For the first demonstration, Heifets’ benchmark molecules (http://www.cs.toronto.edu/~aheifets/ChemicalPlanning/BENCHMARK.tar.gz ) [34] were used to show the scale of the chemical reaction networks constructed by CompRet and the synthetic route enumeration for the networks. The top 100 reaction templates of the prepared template data were used, and *md* was set to six. All calculations were conducted using a single CPU core (Intel(R) Xeon(R) CPU E5-2690 v3 @ 2.60GHz) with 256 GB of RAM.

Table 1 shows the results of the construction of chemical reaction networks and enumerations for the target molecules.

**Table 1:** Experimental result of chemical reaction network construction and synthetic route enumeration for Heifets’ benchmark molecules

Target molecule	The number of OR nodes	The number of AND nodes	The number of edges	The number of synthetic routes	Memory size of the network	Memory size of total routes	Construction time (sec)	Enumeration time (sec)
	29	61	149	70	10.9 KB	58.6 KB	2.68	$2.91\times 10^{(-2)}$
	66	153	405	781	27.5 KB	1.15 MB	6.02	0.185
	8	11	25	8	2.11 KB	7.33 KB	2.96	$9.26\times 10^{(-4)}$
	22	29	58	32	5.38 KB	22.9 KB	2.57	${9.01}\times {10^{(-3)}}$
	92	376	1040	127707	62.6 KB	311 MB	11.7	63.5
	34	78	175	108385	13.1 KB	416 MB	6.33	97.6
	17	19	42	12	4.00 KB	6.77 KB	4.30	${6.20\times 10^{(-4)}}$
	38	58	143	67	11.0 KB	64.5 KB	3.84	0.146
	74	213	567	5213	36.4 KB	9.19 MB	6.35	0.645
	45	93	208	1042	16.3 KB	178 MB	27.5	0.393
	65	139	359	2520	25.9 KB	5.10 MB	9.85	0.346

The numbers of OR and AND nodes indicate the corresponding number of nodes contained in the constructed chemical reaction network. The number of synthetic routes represents the number of enumerated possible routes in the network. The memory sizes of the network and total routes are calculated by converting objects into DOT files. The construction time indicates the computation time required for the construction of the network. The enumeration time indicates the computation time required to enumerate all possible synthetic routes from the network

In Table 1, benchmark molecules whose synthetic routes were designed by CompRet using the top 100 reaction templates are shown. Note that the template set prepared in their study [34] consisted of 50 reactions that were selected to be suitable for synthesis of the benchmark molecules, although they succeeded in finding synthetic routes for most molecules in the benchmark. In Table 1, the second and third columns indicate the number of constituent OR and AND nodes, respectively, that is, the size of the chemical reaction network for the molecule depicted in the first column. The sizes of the generated chemical reaction networks differed significantly between the molecules. The fourth column from the left shows the number of enumeration results of the synthetic routes extracted from each chemical reaction network. More than 100,000 synthetic routes have been successfully enumerated for the fifth and sixth molecules. This number may appear excessive considering the molecules; however, as reported in prior studies [27, 28], the number of synthetic routes can reach $\approx 10^5$ depending on the molecules. Thus, the obtained results are consistent with previous findings. The fifth and sixth columns denote the memory sizes of the constructed network and enumerated routes, respectively. Each object is converted into DOT format [48] to calculate the total amount of memory. The seventh and eighth columns show the generation time of the chemical reaction network and the calculation time of enumeration, respectively. It can be seen that the time for construction tends to be much longer than that for the enumeration of the chemical reaction network. This is because searching for applicable templates for a molecule, and then, using them to divide it into its substances are time-consuming tasks. Besides, enumeration from a larger and more complex network tends to require more time because the number of synthetic routes in a chemical reaction network increases combinatorially. It should be noted that the benchmark originally consists of 20 molecules, half of which remain unsolved. This would be because the reaction templates we used did not include the reactions or starting materials needed to solve the problems (Additional file 1).

### Route recommendation for cetirizine

**Fig. 5:**
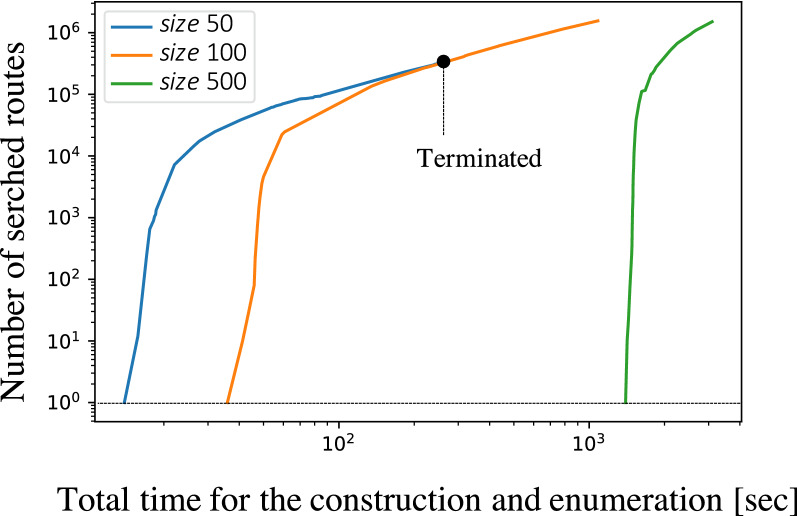
Searched synthetic routes for cetirizine with different template sets. The number of the routes for each chemical reaction network is counted at several time points. The labels *size* 50, *size* 100, and *size* 500 respectively indicate that the top 50, 100, and 500 reaction templates have been utilized. Counting is aborted when the total number of routes exceeds 1,500,000. The dotted line at the bottom indicates that the first synthetic route has been discovered

To examine the synthetic routes designed by CompRet in detail, we have applied CompRet to cetirizine, a drug whose reported synthetic route is relatively simple [49, 50]. Here, the results of changing the template set size and the maximum depth *md* are shown, followed by the routes recommended by CompRet using three scoring methods: REF, MSCS, and STEP. We also performed additional experiments for several molecules; the results are shown in Fig. S5 in Additional file 1. First, the construction of the chemical reaction network and the synthetic routes for different template set sizes (top 50, 100, and 500) were investigated. The value of *md* was fixed to six. Figure 5 shows the time taken for the network constructions. The dotted line indicates that the first route has been found. Finding a single route for the top 100 (orange line) and 500 (green line) template cases required an extended period of time, because the number of candidate routes increased exponentially with the increase in the number of templates. The blue line shows the result for size 50. In this case, the network construction was completed in approximately 30 seconds. In the cases of size 100 and 500, the number of routes increased significantly; thus, enumeration was halted when the number of routes exceeded 1,500,000. In the case of size 500, the time taken to find 1,500,000 routes was less than 2,000 seconds.

**Fig. 6:**
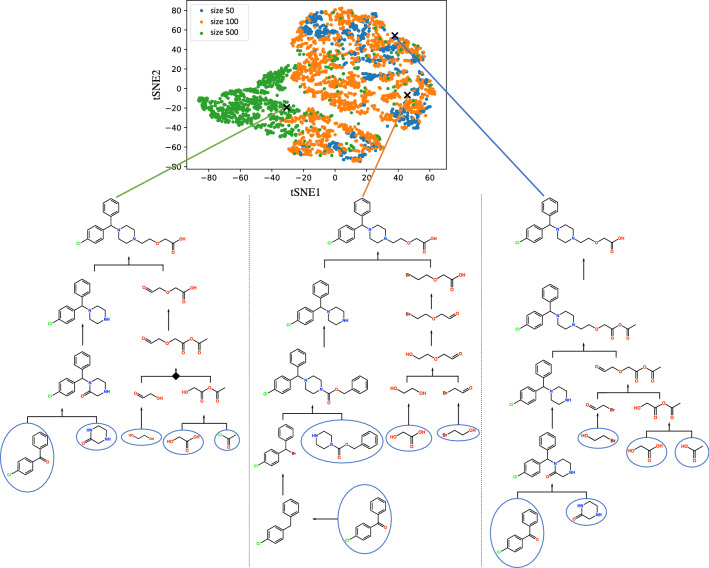
Sampled route distribution by t-SNE embedding. The blue, orange, and green points denote sampled routes from the network constructed with the top 50, 100, and 500 reaction templates, respectively. *md* is fixed to 6. For each setting, 1,000 routes are sampled out of millions of candidates. The black rhombus indicates a chemically unreasonable reaction. Such a reaction may sometimes occur because CompRet algorithmically enumerates synthetic routes

Figure 6 shows the scattered routes with the t-SNE embedding of the obtained synthetic routes, respectively. For ease of viewing, 1,000 randomly sampled routes are shown for each template set size. Three synthetic routes sampled from the distant plot at the top of Fig. 6 have different respective starting materials and reactions. The distribution of the synthetic routes designed for size 50 does not seem to be a subset of those for size 100 because the total numbers of designed routes are different, but the sampled size is the same. Additionally, the precursors of cetirizine in the middle and left routes contain carboxylic acid, while the precursor in the reported route [50], shown as the black sphere route in Fig. 8, contains carboxylate ester.

**Fig. 7:**
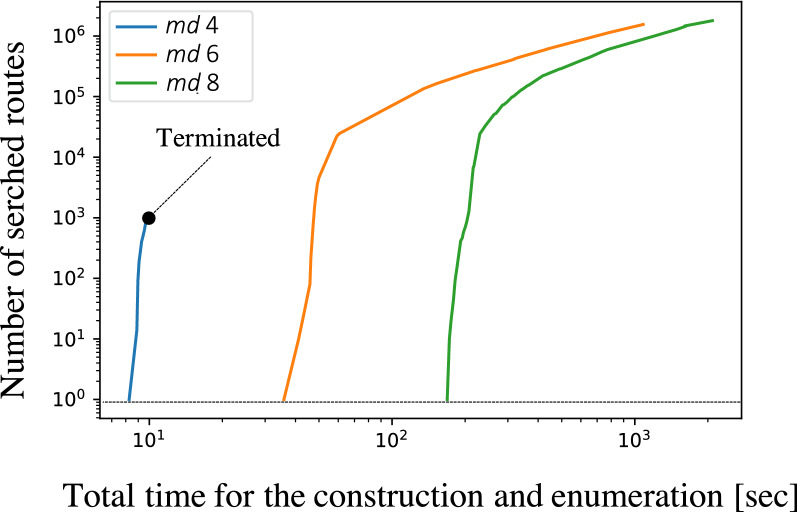
Searched synthetic routes for cetirizine are enumerated, and the number of the routes for each chemical reaction network is counted at several time points. The value of *md* shows the maximum depth of the search algorithm. Counting is aborted when the total number of routes exceeds 1,500,000. The dotted line at the bottom indicates that the first synthetic route has been discovered

The results of constructing the chemical reaction network with the respective *md* values of 4, 6, and 8 are shown in Fig. 7. The top 100 reaction templates were used throughout. The blue line in Fig. 7 is the result for *md* = 4, where the chemical reaction network was constructed in less than 10 seconds, and 853 routes were obtained. The orange line is identical to that in Fig. 5, that is, the result of construction with *md* = 6. Enumeration was halted when the number of routes exceeded 1,500,000. Figure S4 in Additional file 1 depicts the scattered routes explored with each *md* using t-SNE embedding and several examples of them. This visualization also shows a variety of routes explored by CompRet.

**Fig. 8:**
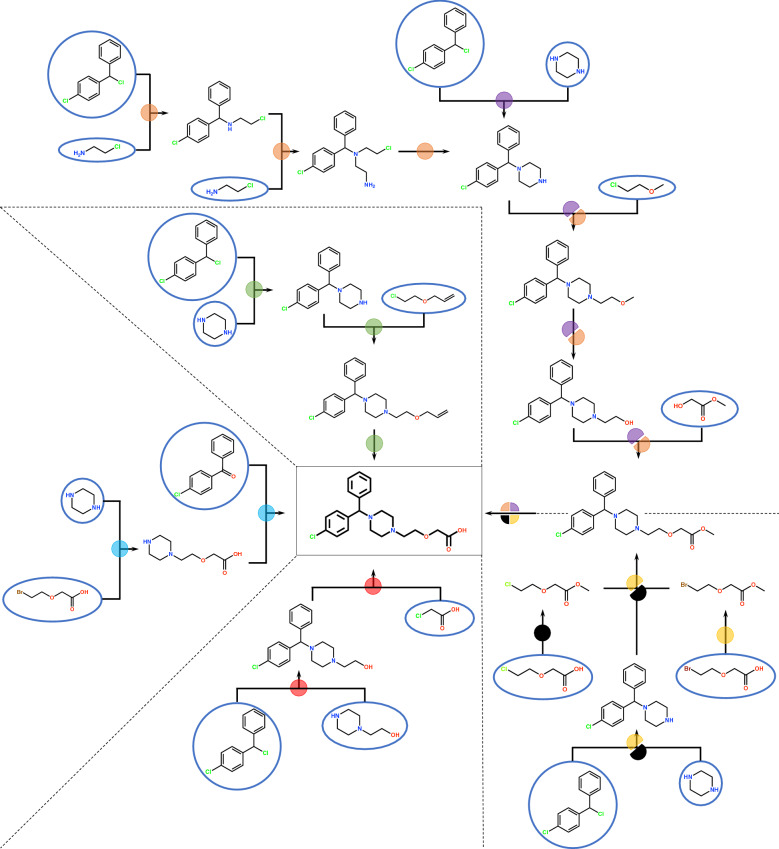
Examples of multiple synthetic routes for cetirizine recommended by CompRet. Each synthetic route is distinguished based on color. Parts of some routes may overlap, denoted by split spheres. In particular, the black sphere depicts the reported routes of cetirizine [50]

Finally, the results of route recommendation using the route scoring methods REF, MSCS, and STEP are shown in Fig. 8. The figure illustrates several examples of synthetic routes recommended by these methods, explored using the top 500 templates and an *md* of 6. We also show the details of REF and MSCS distributions in Fig. S2 and S3, and Table S1 and S2 in Additional file 1. Compret successfully obtained the synthetic route reported in the literature [50], denoted by the black sphere in Fig. 8. Using this known route as the reference for the REF methods, CompRet recommended the yellow route that changed chloride to bromine in the reference route. Its similarity score was the highest (6.32) among the enumerated routes. This result indicates that the REF method is suitable for finding routes similar to the reference route. Using MSCS, CompRet recommended the orange and purple routes, whose MSCS scores were 1.80 (lowest) and 2.28, respectively. The two routes are different from the reference route; thus, the MSCS method would have the potential to provide a variety of synthetic routes. Using STEP, CompRet recommended the red and light blue routes, whose number of steps was the smallest. The green route also has a smaller number of synthetic steps. While these routes had smaller numbers of synthetic steps, their starting materials and reactions differed. Consequently, the recommendation of routes that differed slightly from the known route, as well as diverse alternatives, demonstrates the effectiveness of the CompRet framework.

## Conclusions

In summary, we developed CompRet, a new recommendation framework for CASP. It consists of three parts: DFPN-based chemical reaction network construction of a given target, enumeration of synthetic routes from a given chemical reaction network, and recommendation from the enumerated synthetic routes. In this study, the DFPN algorithm, which was employed to search for synthetic routes [34], is extended to construct a chemical reaction network. Furthermore, we have mathematically proven the validity of the enumeration algorithm. Since a chemical reaction network is built for the target compound each time, synthetic routes can be designed for the new compound as well. This algorithm works in general for a given appropriate chemical reaction network with a mathematical guarantee. CompRet was also applied to Heifets’ benchmark molecules and cetirizine. It was demonstrated to be able to construct chemical reaction networks, containing over a million routes in some cases, with a relatively small computational cost. Furthermore, the recommendation and visualization methods could be useful to suggest a wide variety of conceivable synthetic routes, from a slight deviation from the existing route to significantly different alternatives.

The current version of CompRet is not at the stage of practical use and has room for improvement as follows. Naively enumerating all synthetic routes leads to exponential growth of a chemical reaction network. Although the exponential growth is essentially difficult to overcome, parallel tree search algorithms [51] have the potential to hand this problem. In-scope filter [18] or other graph pruning algorithms [21] will also provide solutions. As shown in the demonstration of chemical reaction network constructions, the explored synthetic routes depend on the template set and maximum depth *md*. The set of starting materials also affects the search results. Basically, CompRet can find a larger number of routes with a larger dataset and deeper depth setting. However, for practical applications, such a dataset and parameters should be determined adaptively based on the given case.

Further, template-based methods possess weaknesses such as computationally expensive subgraph isomorphism calculation [52]. In the future, it may be effective to construct chemical reaction networks by using refined template extraction methods [20], user-defined reaction templates, and template-free methods [9, 11, 52–54]. Although CompRet employs Reactor for retrosynthetic computation, other engines, such as RDChiral [55], ASKCOS [19] and RTSA [15], would be effective alternatives. This study only considered simple methods for route recommendation. Designing synthetic route evaluation metrics that are effective in all situations is a challenging task because critical aspects of route design depend on the chemist’s objectives and/or needs [56]. However, various route evaluation methods, including SCScore with reformulation [12, 21] and other deep learning-based methods [57], have been proposed in recent years. If these methods are appropriately combined with CompRet, the customized framework could function as a user-friendly route recommendation system. The results obtained in this work are considered to be a successful example of bridging CASP and the field of discrete mathematics and developing an enumeration algorithm from a new perspective. This can enable the practical improvement of CASP through algorithmic techniques. Finally, to improve usability of the framework, we plan to provide the Docker image and the web application of CompRet.

## Availability and requirements

- Project name: CompRet
- Project home page: https://github.com/fullswing/CompRet
- Operating system(s): Platform independent
- Programming languages: Java, Python
- Other requirements: ChemAxon

## Supplementary information

**Additional file 1.** CompRet: a comprehensive recommendation framework for chemical synthesis planning with algorithmic enumeration. **Figure S1**: Illustration of (**a**) basic depth-first proof number search and (**b**) procedures to continue searching after finding a proof tree. **Figure S2**: Distribution of the REF scores of the found synthetic routes for cetirizine. **Figure S3**: Distribution of the MSCS scores of the found synthetic routes for cetirizine. **Figure S4**: Examples of sampled routes' t-SNE embedding. **Table S1**: Detailed information of the REF distribution. **Table S2**: Detailed information of the MSCS distribution.

## Data Availability

The data extracted from industrial datasets from Reaxys, cannot be provided to the public. The source code of the network construction and enumeration algorithm is available at https://github.com/fullswing/CompRet.
