# Supplementary material for: CompRet: a comprehensive recommendation framework for chemical synthesis planning with algorithmic enumeration
Source: J Cheminform. 2020 Sep 1;12:52. doi: 10.1186/s13321-020-00452-5 (PMC7465358; doi:10.1186/s13321-020-00452-5)
Supplement: Supplementary file 1 — Additional file 1. CompRet: a comprehensive recommendation framework for chemical synthesis planning with algorithmic enumeration. Figure S1: Illustration of (a) basic depth-first proof number search and (b) procedures to continue searching after finding a proof tree. Figure S2: Distribution of the REF scores of the found synthetic routes for cetirizine. Figure S3: Distribution of the MSCS scores of the found synthetic routes for cetirizine. Figure S4: Examples of sampled routes' t-SNE embedding. Table S1: Detailed information of the REF distribution. Table S2: Detailed information of the MSCS distribution. [file 13321_2020_452_MOESM1_ESM.pdf]

Supporting Information for "CompRet: a comprehensive recommendation framework for chemical synthesis planning with algorithmic enumeration"

Ryosuke Shibukawa,[†,△] Shoichi Ishida,[‡,△] Kazuki Yoshizoe,[¶] Kunihiro Wasa,[§] Kiyosei Takasu,[‡] Yasushi Okuno,[‖,⊥] Kei Terayama,[*,#,¶,‖,⊥] and Koji Tsuda[*,†,@,¶]

†*Graduate School of Frontier Sciences, the University of Tokyo, Kashiwa, 277-8561, Japan*

‡*Graduate School of Pharmaceutical Sciences, Kyoto University, Sakyo-ku, 606-8501, Japan*

¶*RIKEN Center for Advanced Intelligence Project, Tokyo, 103-0027, Japan*

§*Toyohashi University of Technology, Toyohashi, 441-8580, Japan*

‖*Graduate School of Medicine, Kyoto University, Sakyo-ku, 606-8507, Japan*

⊥*Medical Sciences Innovation Hub Program, RIKEN, Yokohama, 230-0045, Japan*

#*Graduate School of Medical Life Science, Yokohama City University, Tsurumi-ku, 230-0045, Japan*

@*Research and Services Division of Materials Data and Integrated System, National Institute for Materials Science, Tsukuba, 305-0047, Japan*

△*These two authors contributed equally*

E-mail: terayama@yokohama-cu.ac.jp; tsuda@k.u-tokyo.ac.jp

Phone: +81 (0)4 71363983. Fax: +81 (0)4 71363982

1 Definition of AND/OR tree and chemical reaction network.

In this study, we utilize a tree structure to represent a synthetic route as shown in Fig.1. A *directed graph* G is a pair of a node set V and a edge set $E \subseteq V \times V$. Let π be an alternating sequence of vertices and edges $\pi = (v_1, e_1, v_2, e_2, \ldots, e_{k-1}, v_k)$ such that for each $i \in \{1, \ldots k-1\}$, $e_i = (v_i, v_{i+1})$. π is a *path* if π consists of distinct vertices. In particular, we call such π a *path from v_1 to v_k*. π is a *cycle* if $v_1, \ldots, v_{k-1}$ are mutually distinct and $v_k = v_1$. G is *connected* if for any node pair u, v in G, there is a path from u to v or from v to u. G is *acyclic* if G has no cycle. A directed acyclic graph G is a *directed tree* if G satisfies the following conditions: (1) there is a unique special node r, called the *root*, in G such that for any node $v \in V$, there is a path from r to v, for each $v \in V$, and (2) there is at most one node $u \neq v$ in V such that $(u, v) \in E$. We call such u the *parent* of v and we say v is a *child* of u. Note that u may have more than one child. A node is called a *leaf* if it has no child. A node is an *interior* node if it is not a leaf. We can see that, in a directed tree, a path from u to v is unique for any node pair u and v. The *depth* of a node v in a directed tree is the number of edges contained in the path from the root to v. A graph $G' = (V', E')$ is a *subgraph* of G if $V' \subseteq V$ and $E' \subseteq E \cap (V' \times V')$ hold. In the following, we give some key notations in this paper.

Definition 1.1. [AND/OR tree] A directed tree T is an *AND/OR tree* if T satisfies the following conditions:

1. Each node is assigned either *AND* or *OR* such that AND and OR appear alternately in all the paths on T.

2. All the leaves are assigned OR.

3. Each node has a *label* whose value is either True, False, or unknown.

4. For each OR node n in T, if there is a child node of n whose label is True, then the label of n is True. Otherwise, the label of n is False.

5. For each AND node n in T, if the labels of all child nodes of n are True, then the label of n is True. Otherwise, the lable of n is False.

Here, an OR and AND node correspond to a molecule (target) and a reaction template, respectively. The root is typically an OR node because the root corresponds to the target compound. A terminal node in an AND/OR tree is defined as a leaf whose label is True or False. Note that, in an AND/OR tree, merging at an OR node is allowed, i.e., an AND node may have more than two parents, but there is no cycle.

Next, we introduce the notion of a proof tree to represent a single synthetic route.

Definition 1.2. [Proof tree] An AND/OR tree T is a proof tree if T satisfies the following conditions.

1. The label of the root node is True.

2. An OR node has at most one child node.

3. Each AND node (i.e., reaction template) has molecules that are required for its corresponding reaction as its children.

The second condition indicates that, for each molecule in a proof tree, just one synthetic route exists to make it.

A chemical reaction network of a given target molecule is represented as a graph structure of multiple synthetic routes (AND/OR trees) as shown in Fig.2. Hence, the third condition implies that to make the label of an AND node n True, we need to pick all the required nodes of n from the chemical reaction network for a target molecule.

Definition 1.3. [Chemical reaction network] A chemical reaction network for a target molecule c is a directed acyclic graph that satisfies the following conditions.

1. All nodes in the network consist of AND nodes and OR nodes.

2. There exists just one root node, which has no parents, and it corresponds to the molecule c.

3. There are no more than two OR nodes that correspond to the same molecule. There are no more than two AND nodes that correspond to the same reaction template.

4. Each AND node has exactly one parent node, while an OR node can have several parent nodes.

5. Each node has its label, which satisfies the 4th and 5th conditions in Def 1.1.

6. All leaf nodes are OR nodes whose labels are True.

2 Construction of chemical reaction network

In this paper, we propose an algorithm to construct a chemical reaction network for a given target molecule using the DFPN algorithm. We refer to this algorithm as Ex-DFPN (Extended DFPN). We show pseudocodes for Ex-DFPN in Algorithm S1 and S2. We implemented the algorithm referring to the DFPN implementation by Nagai .[1,2] To perform the DFPN-based search, we use a variant of the AND/OR tree with additional properties. In the tree, each node has the following values:

- Proof number (pn)
- Disproof number (dn)
- pn threshold (pnTh)
- dn threshold (dnTh)

and each edge has edge cost (e).

First, we explain the basic procedure of DFPN. We described the algorithm explained in this paragraph as pseudocode in Algorithm S1. DFPN consists of three steps (Select, Expand, and Update) illustrated in Fig. S1 (a). First of all, DFPN selects a node to expand based on an evaluation function. We will explain the details of the evaluation function later. In the Expand step, DFPN expands the selected node by computing reactants using reaction templates. When DFPN visits an OR node for the first time, it checks whether the molecule is commercially available or not. If the molecule is found, then the values of the evaluation functions of the node are set to 0 and DFPN returns to the parent node. Also, DFPN returns when the depth of the node reaches the depth threshold, which is a parameter to restrict the search space. In the Update step, DFPN updates the value of the evaluation function for the node.

We developed Ex-DFPN for constructing chemical reaction networks by adding some procedures that make the basic DFPN algorithm continue searching even after finding a

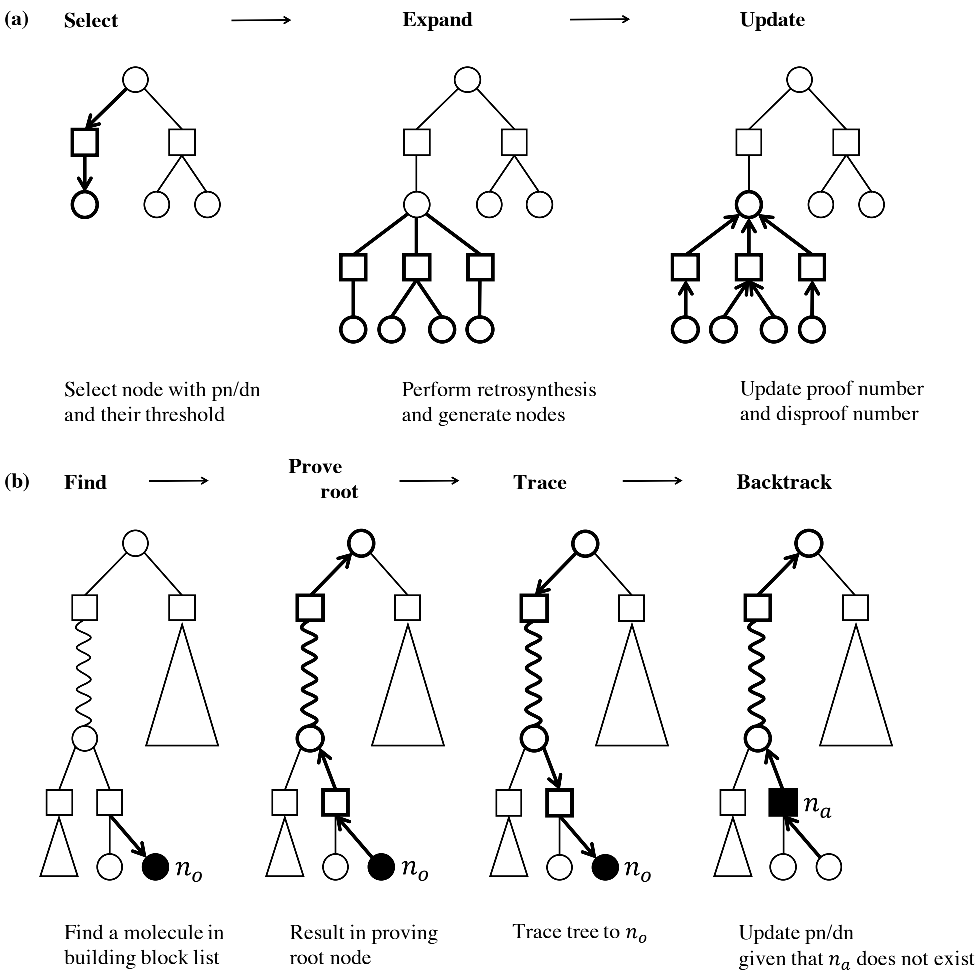

Figure S1: Illustration of (a) basic depth-first proof number search and (b) procedures to continue searching after finding a proof tree. Circle and square represent OR and AND nodes, which denote a molecule and a reaction template respectively.

solution. This procedure is implemented in the traceProofTree function, called in line 34 of Algorithm S1 and described in Algorithm S2. After finding a solution, Ex-DFPN performs some additional procedures illustrated in Fig.S1.(b) to continue searching. The basic idea of these procedures is to continue searching by assuming that the newly found solution does not exist. When Ex-DFPN finds a solution, there always exists a last found AND-node or OR-node. After determining the node in the trace step, Ex-DFPN updates the value of evaluation functions for nodes in the pathway from root, assuming that the node does not exist. With these procedures, Ex-DFPN can construct a chemical reaction network for a given target molecule.

We designed evaluation functions based on the proof number and disproof number. A proof number denotes the number of nodes to prove to show the node is true. A disproof number denotes the number of nodes to disprove to show the node is false. Because proof/disproof numbers for OR nodes and AND nodes have duality, we can simply define them as below.

$$n.\phi = \min(\{n_c.\delta | n_c \in n.\text{children}\}) \tag{1}$$

$$n.\delta = \text{sum}(\{n_c.\phi | n_c \in n.\text{children}\}) \tag{2}$$

where $n.\phi$ denotes proof number and $n.\delta$ denotes disproof number for OR node n, while the contrary holds for AND node. In addition, we utilized tree height information. Tree height is calculated for each node denoting the number of edges to the descendant leaf node. Using this evaluation, Ex-DFPN prefers synthetic routes with smaller steps.

Algorithm S1 retrosynthesis using depth first proof number search

```
function orSearch(node)                                   ▷ Search OR node
    if mol is commercially available then
        node.φ ← 0
        node.δ ← ∞
        return
    end if
    moleculeTableLookUp(node)
    if node.φ ≤ node.Φ  &  node.δ ≤ node.Δ then
        node.Φ ← node.φ
        node.Δ ← node.δ
        return
    end if
    if node has not expanded yet then
        expandNode(node)
    end if
    if node has no child node then
        if mol is commercially available then
            node.φ ← 0
            node.δ ← ∞
        else
            node.δ ← 0
            node.φ ← ∞
        end if
        moleculeTableSave(node) return
    end if
    while node.Φ > node.deltaMin()  &  node.Δ > node.phiSum() do
        n_c, φ_c, δ_2 = selectNode(node)
        n_c.Φ ← node.δ + φ_c − n.phiSum()
        n_c.Δ ← min(node.φ, δ_2 + 1)
        andSearch(n_c)
        if node.deltaMin() = 0 then
            if all children are proven to be true then continue
            end if
            traceProofTree(root)
            delete(bestChild)
        end if
    end while
    node.φ ← node.deltaMin()
    node.δ ← node.phiSum()
    moleculeTableSave(node)
end function
```

```
function andSearch(node)                                        ▷ Search AND node
    if node has not expanded yet then
        node.delta ← node.children.size
    end if
    if node has only a child node then
        OrSearch(child_node)
    else
        while node.φ > node.deltaMin()  &  node.δ > node.phiSum() do
            n_c, φ_c, δ_2 = selectNode(node)
            n_c.φ ← node.δ + φ_c − n.phiSum()
            n_c.δ ← min(node.φ, δ_2 + 1)
            orSearch(n_c)
        end while
    end if
    node.φ ← node.deltaMin()
    node.δ ← node.phiSum()
end function
function expandNode(node)                                       ▷ Expand OR node
    for reaction in reactionDatabase do
        products ← computeRetrosynthesis(node.mol, reaction)
        if products != null then
            r ← andNode(reaction)
            node.children.add(r)
            for m in products do
                key ← molToSmiles(m)
                n ← orNode(m, node)
                if !nodeHashMap.contains(key) then
                    nodeHashMap.put(key, n)
                    r.add(n)
                else
                    prev ← nodeHashMap.get(key)
                    if prev is node's ancestor then             ▷ Care for cycle
                        delete(r)
                        break to outer loop
                    else
                        r.add(prev)
                    end if
                end if
            end for
        end if
    end for
end function
```

```
function moleculeTableSave(node)
    key ← molToSmiles(node.mol)
    nodeHashMap.put(key, node)
end function
function moleculeTableLookUp(node)
    key ← molToSmiles(node.mol)
    if nodeHashMap.contains(key) then
        node.Φ ← nodeHashMap.get(key).φ
        node.Δ ← nodeHashMap.get(key).δ
    else
        node.Φ ← 1
        node.Δ ← 1
    end if
end function
```

Algorithm S2

```
function tracePROOFTREE(node)
    pathFromRoot.add(node)
    if node.lastSelectNodeIndex ! = -1 then
        next ← node.children.get(node.lastSelectNodeIndex)
        tracePROOFTREE(next)
    else
        lastFoundNode ← pathFromRoot.get(pathFromRoot.size-1)
        flagProofTree ← false
        if lastFoundNode.getProofNumber() = 0 then
            flagProofTree ← true
        end if
        lastFoundAndNode ← pathFromRoot.get(pathFromRoot.size-2)
        for n in pathFromRoot do
            if n is AND node & n.isPartOfProofTree = false then
                sideRoute ← new ArrayList sideRoute.add(n)
                for nc in n.children do
                    if nc is not a last Selected node then
                        sideWALK(n.children.get(j), sideRoute)
                    end if
                end for
            end if
        end for
        lastFoundAndNode.ignore ← true
        for n in pathFromRoot do
            n.isPartOfProofTree ← true
        end for
        if flagProofTree then
            for n in lastFoundAndNode.children do
                n.isPartOfProofTree ← true
            end for
        else
            lastFoundAndNode.isPartOfProofTree ← false
        end if
    end if
    if node.children.size() > 0 then
        node.φ ← deltaMIN(node)
        node.δ ← phiSUM(node)
    end if
end function
```

```
function sideWalk(node, sideRoute)
    node.isPartOfProofTree ← true
    if node.lastSelectNodeIndex ! = -1 then
        next ← node.children.get(node.lastSelectNodeIndex)
        sideRoute.add(next)
        if node.getProofNumber() = 0 then
            node.isPartOfProofTree ← true
        end if
        sideWalk(next, sideRoute)
        if node.children.size() > 0 then
            node.φ ← deltaMin(node)
            node.δ ← phiSum(node)
        end if
    end if
end function
```

3 Synthetic Route Enumeration algorithm

In this section, we introduce an enumeration algorithm, called the SRE (Synthetic Route Enumeration) algorithm, that lists all synthetic routes for a given chemical reaction network. The SRE algorithm takes a chemical reaction network as input and outputs all synthetic routes contained in the network without duplication. There are several techniques in the field of enumeration algorithm: backtrack, reverse search, Glay code, and partition.[3–6] While each technique has advantages and disadvantages, we have employed the partition technique for designing the SRE algorithm because it is simple and can efficiently prune the search space.

The procedure of our SRE algorithm is illustrated in Fig. 4 and the pseudocode is shown in Algorithm S3. Our SRE algorithm enumerates all synthetic routes by recursively partitioning the set of solutions into two disjoint subsets. Firstly, the algorithm picks the root node, which is node 1 in Fig. 4, and divides the set of solutions into two disjoint set; the one consists of the solutions containing node 1 and the other consists of the remaining solutions. Next, the algorithm enumerates all the solutions containing node 1 by focusing on node 3 and dividing the set into two disjoint sets again, i.e., the one consists of the solutions containing both nodes 1 and 3, and the other consists of the solutions containing node 1 but not node 3. The SRE algorithm repeats this procedure recursively for all nodes. When the SRE algorithm terminates, it is ensured that all synthetic routes are enumerated. Note that when the algorithm enumerates solutions containing node 1 but not containing node 5, node 6 must be contained in all solutions, and thus the algorithm can skip the case of containing node 1 and not containing nodes 5 or 6. This pruning yields the effectiveness of the algorithm. The proof of this guarantee is given in the next section.

Algorithm S3 Enumeration

```
G: Directed graph (no cycles)
P ← {}: Prohibited list of and nodes
A: And node set
O: Or node set
V ← A ∪ O: Node set
R ← {∀v ∈ O | G.out_degree(v) = 0 ∧ v.bool = True}: Terminal node set
ENUMERATE()
function ENUMERATE()
    g ← Graph()
    g.addNode(root)
    PARTITION(g, root)
end function
function PARTITION(g, n :or node)
    if n ∈ R then
        output(g)
    end if
    neighborsToSearch ← {∀v ∈ A | v ∈ G.neighbors(n) ∧ v ∉ P}
    if neighborsToSearch is empty then
        for k in G.neighbors(n) do
            P ← P \ {k}
        end for
        return
    end if
    g_ ← copy(g)
    p ← neighborsToSearch.get(0)
    g.addNode(p)
    g.addEdge(n, p)
    for o_i in G.neighbors(p) do
        g.addNode(o_i)
        g.addEdge(p, o_i)
    end for
    P ← P ∪ {p}
    leafNodes ← {v | v ∈ g.leafNodes∧ v ∉ R}
    if leafNodes is empty then
        output(g)
    else
        t ← leafNodes.get(0)
        PARTITION(g, t)
    end if
    PARTITION(g_, n)
end function
```

4 Proof of completeness and soundness of the SRE algorithm

In this section, we prove that the SRE algorithm can list all synthetic routes in a given chemical reaction network without duplication. To prove this, we need to show the following: the output does not include invalid synthetic routes or any duplication, and the output of the algorithm includes all the synthetic routes (proof trees) in a chemical reaction network. We call the former property the soundness and the latter property the completeness of the algorithm.

To show the soundness, we first prove that no duplication appears in the output of the algorithm.

Lemma 4.1. There is no duplication in the output of the SRE algorithm.

Proof. Let $R_1 = partition(g_1, t_1), R_2 = partition(g_2, n_2)$ be two different recursions. It is enough to show that these two recursions output different solutions. We consider the following cases:

<u>R_1 is an ancestor or a descendant recursion of R_2:</u> Without loss of generality, we can assume that R_1 is an ancestor of R_2. The output of R_1 includes p by line 26 of Algorithm S3, while g_2 of R_2, which is called under recursion in line 40, never includes p. By this observation, R_2's output never includes p. Therefore, outputs of R_1 and R_2 are different from each other.

<u>Otherwise:</u> Let R_j be a common ancestor of R_1 and R_2. Such R_j always exists because R_1 and R_2 are not the root recursion. In lines 38 and 40 of Algorithm S3, R_j generates two child recursions whose input graphs g and g_- are different. Therefore, outputs of R_1 and R_2 are different.

Finally, different recursions always output different proof trees. □

We then show the soundness of the SRE algorithm.

Theorem 4.1. *The SRE algorithm has soundness.*

Proof. From Lemma 4.1, there are no duplicates in the outputs of Algorithm S3.

What remaining to show is that the output only contains correct solutions. Because n in Algorithm S3 is an OR node, p is an AND node. From line 26-31 in Algorithm S3, all interior OR nodes in g have exactly one child OR node, while AND nodes have all child OR nodes. Once an AND node is added as a child node of an OR node, then the OR node is no longer a leaf node. Therefore, no AND node is added as a child node of an OR node. From line 33, all leaf nodes in a tree are terminal nodes, and thus, from the property of an AND/OR tree, the label of the root node is also True. □

Finally, we prove that Algorithm S3 outputs all the solutions.

Theorem 4.2. *The SRE algorithm has completeness.*

Proof. If *root* is a terminal node, then by line 15, the solution is outputted. Hence, in what follows, assume that *root* is not a terminal.

We say that Q is a *partial proof tree of* T, abbreviated as a ppt of T, if Q is a connected subgraph of T such that all the leaves in Q are assigned OR and the root of Q is the root of T. Let $s(Q)$ be the number of AND nodes in Q.

We prove, by induction on $s(\cdot)$, the following statement: For any ppt Q with k AND nodes, Algorithm S3 makes a recursive call (Q', n) such that Q' is a ppt of Q, Q' has $k-1$ AND nodes, and n has an AND neighbor in $Q \setminus Q'$. If this statement holds, then we see that for any ppt Q, there is a recursive call in which $g = Q$ after line 31. In addition, clearly, a proof tree is also a ppt. A proof tree has no leaf which is not a terminal node. Hence, because of line 34 and because Algorithm S3 makes the recursive call for an empty ppt in line 11, all proof trees are outputted.

Clearly, when the size is one, that is, for a ppt for the root node, the statement holds by line 11. Next we assume that for any ppt Q with k AND nodes, the hypothesis holds. Let

S be a ppt such that $s(S) = k + 1$ and Q is a ppt of S.

From the assumption, there is a ppt Q' of Q such that Algorithm S3 makes a recursive call C which receives Q' and an OR node n satisfying $s(Q') = k - 1$ and n has an AND neighbor q in $Q \setminus Q'$. In the remainder, we show that there is a recursion call which receives Q and a desired AND node n'.

If q is not in P, then clearly, there is some descendant recursive call C' that satisfies the hypothesis. Suppose that q is in P. This implies that there is an ancestor recursive call C' of C such that during the execution of C', q is added to P. Note that n is the unique parent for q from the definition of input graphs. Hence, from line 17, C' receives n as an input and C' makes a recursive call C'' which receives Q.

Let n' be the parent of p', where p' is an AND node in $S \setminus Q$. Because Q is a ppt for S, such n' and p' always exist. Note that S must contain n'. If n' is not in Q, then there is no AND node p^* such that $(p^*, n') \in Q$. Because n' is in S, this contradicts that p' is the only AND in $S \setminus Q$. Suppose that n' is not a leaf in Q. However, this creates a contradiction because n' is a leaf in $S - p'$. Hence n' is a leaf in Q. Because (n', p') is a unique incoming edge to p, by a similar argument for p, there is an descendant recursive call of C'' receiving Q and n' such that p' is not in P. Hence, the statement holds. □

5 Additional information for cetirizine experiments

In this section, distributions of REF and MSCS scores of the found synthetic routes with different sizes of reaction template-sets are provided. Also, examples of synthetic routes found by CompRet with different *md* are shown. Figure S2 shows a distribution of REF for randomly sampled synthetic routes. In cetirizine's case, the maximum valued of REF is 7.0, showing that the route is the same as the route reported in the literature. Mean, variance, and random sampling details are shown in Table S1. For REF score, the distribution shows that routes selected based on REF in Figure 8 are better than other synthetic routes. For MSCS score, the distribution shows that routes selected based on MSCS in Figure 8 are better than the mean value.

Table S1: Detailed information of the REF distribution. The parameter *md* is fixed at 6. Because REF for most synthetic routes are 0, only routes with nonzero REF are in the distribution. With $md = 6$ and $size = 50$, for example, a total of 64,699 routes are randomly sampled from a constructed chemical reaction network and the number of synthetic routes with nonzero REF value is 470. We normalized the values of 470 synthetic routes for distribution then plotted them.

	size 50	*size* 100	*size* 500
the number of routes with nonzero REF	470	611	348
total	64,699	370,255	600,062
mean	3.17	3.25	3.15
variance	1.66×10^{-1}	3.23×10^{-1}	8.08×10^{-2}

Figure S3 shows distributions of MSCS scores for randomly sampled synthetic routes designed with different sizes of the template sets. To compare the effect of template sizes, each distribution is shown as a probability density. Table S1 summarises the mean and variance values of the distributions. As shown in Fig. S3, MSCS scores tend to decrease as the template size increases. This result suggests that the different template sizes may change the number of transformation patterns, and consequently, the tendency of the obtained synthetic routes.

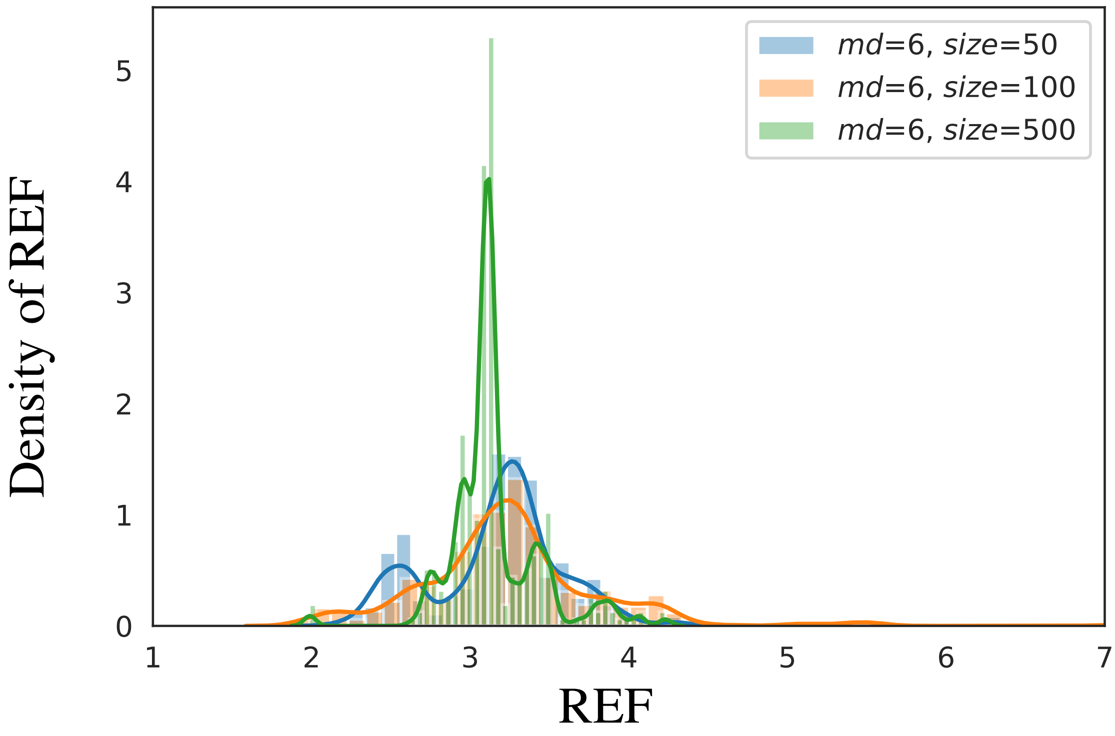

Figure S2: Distribution of the REF scores of the found synthetic routes for cetirizine. The parameter md is fixed at 6. The parameter $size$ denotes the size of reaction templates. Because the REF values for most found synthetic routes are 0, only routes with nonzero REF values are shown in the distribution. Synthetic routes are scored after random sampling, and then normalized.

Table S2: Detailed information of the MSCS distribution. The parameter md is fixed at 6. In each case, 10,000 synthetic routes are sampled and then scored.

	$size$ 50	$size$ 100	$size$ 500
mean	2.16	1.96	3.14
variance	2.35×10^{-2}	4.19×10^{-2}	2.5×10^{-2}

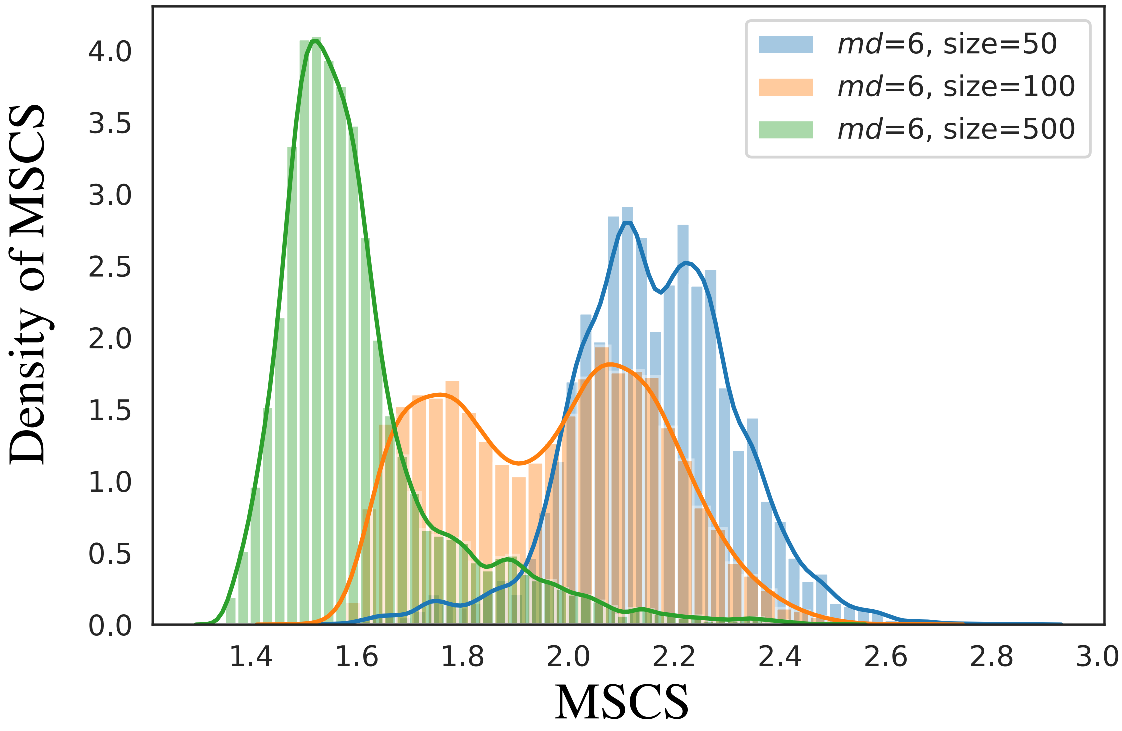

Figure S3: Distribution of the MSCS scores of the found synthetic routes for cetirizine. The parameter md is fixed at 6.

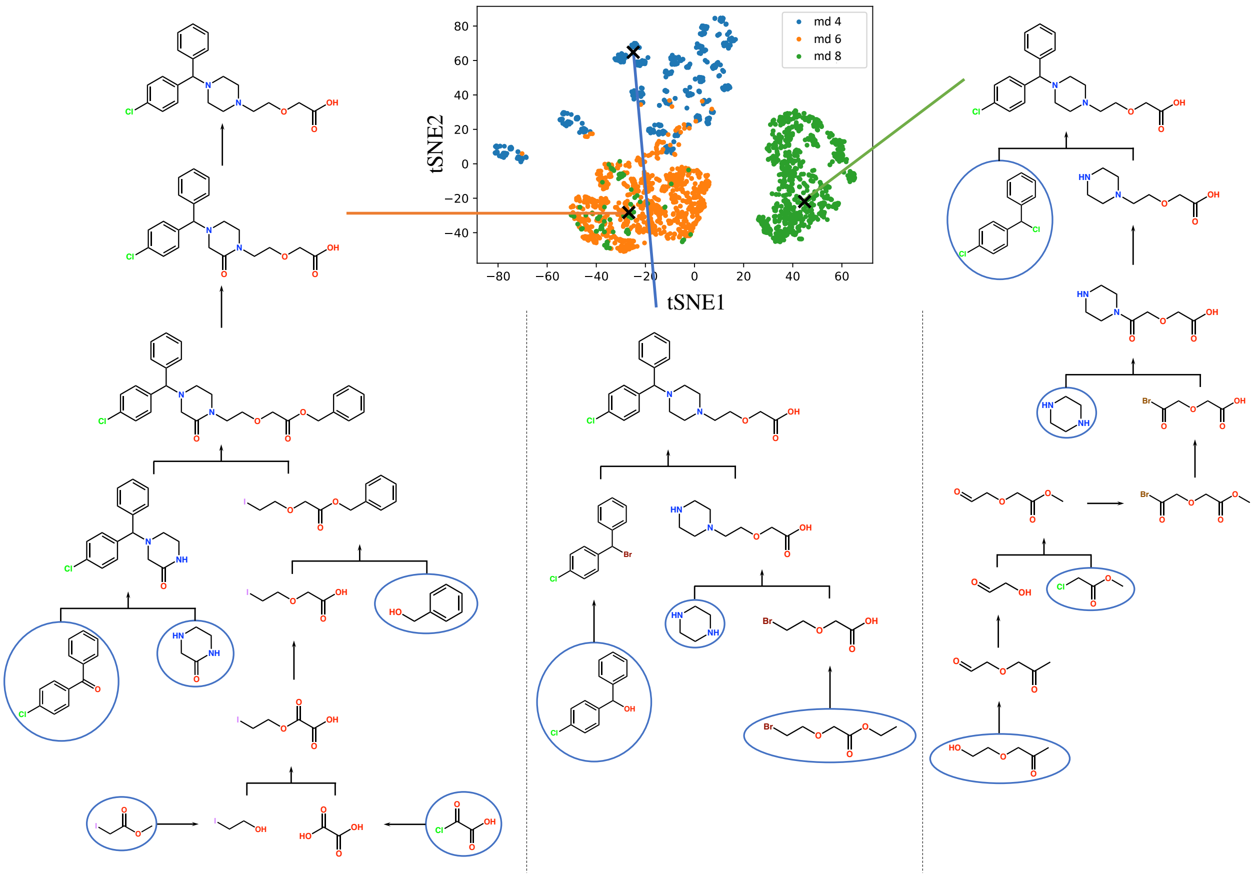

Figure S4: Examples of sampled routes' t-SNE embedding. Blue points denote sampled routes from the network constructed with $md = 4$, while orange for $md = 6$ and green for $md = 8$. The reaction templates are fixed to frequent top 100. For each setting 1000 routes are sampled out of millions of candidates, except for the case that $md = 4$, where the total number of designed routes is 853.

6 Additional experiments

We performed additional experiments on quinaprilat, whose size is larger than cetirizine, and a molecule reported in Segler et al.[7] Figure S5 shows examples of designed routes for each molecule, selected based on STEP.

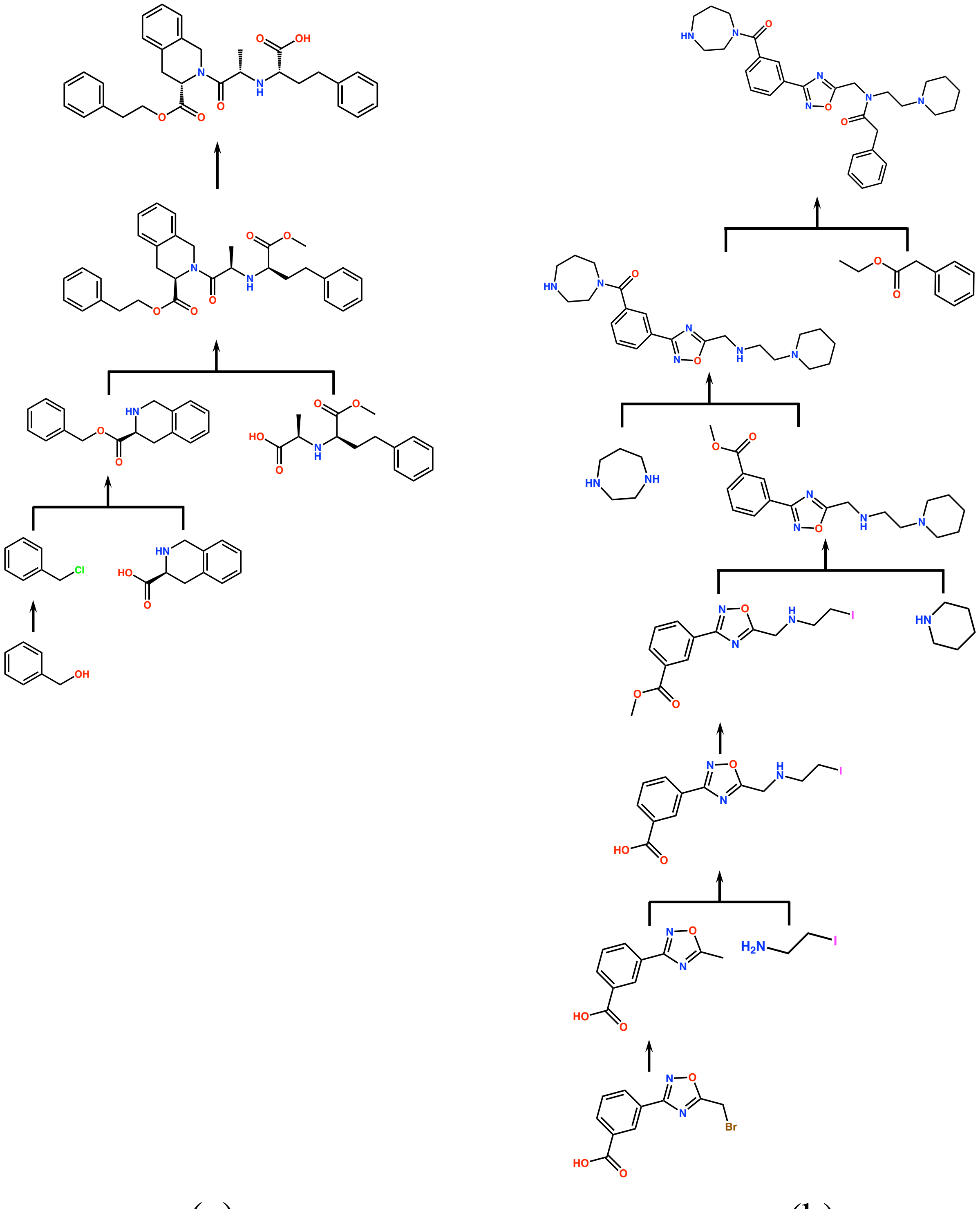

(a) (b)

Figure S5: (a) An example of synthetic routes for quinaprilat. (b) An example of synthetic route for a molecule (id = 1603272692) reported in Segler et al.[7]

References

(1) Nagai, A. Application of df-pn+ to Othello endgames. Proceedings of Game Programming Workshop'99. 1999.

(2) Nagai, A. Df-pn algorithm for searching AND/OR trees and its applications. *PhD thesis, Department of Information Science, University of Tokyo* **2002**,

(3) Minty, G. A simple algorithm for listing all the trees of a graph. *IEEE Transactions on Circuit Theory* **1965**, *12*, 120–120.

(4) Read, R. C.; Tarjan, R. E. Bounds on backtrack algorithms for listing cycles, paths, and spanning trees. *Networks* **1975**, *5*, 237–252.

(5) Savage, C. A survey of combinatorial Gray codes. *SIAM review* **1997**, *39*, 605–629.

(6) Marino, A. *Analysis and Enumeration*; Atlantis Press: Paris, 2015.

(7) Segler, M. H.; Preuss, M.; Waller, M. P. Planning chemical syntheses with deep neural networks and symbolic AI. *Nature* **2018**, *555*, 604.
